# Profiling endothelial function, oxidative stress and inflammatory biomarkers in individuals with various cardiovascular risk factors: the African-PREDICT study

**DOI:** 10.1038/s41440-025-02392-9

**Published:** 2025-10-15

**Authors:** Adriaan Jacobs, Ruan Kruger, Wayne Smith, Maserame C. Mokhaneli, Catharina M. C. Mels

**Affiliations:** 1 Hypertension in Africa Research Team (HART), North-West University, Potchefstroom, South Africa; 2 MRC Research Unit for Hypertension and Cardiovascular Disease, North-West University, Potchefstroom, South Africa

**Keywords:** Adiposity, cardiovascular disease, cholesterol, inflammation, young adults

## Abstract

Cardiovascular risk factors are known to contribute to cardiovascular disease (CVD) development by inducing endothelial activation, increasing oxidative stress and pro-inflammation. The early interaction between these pathways and individual cardiovascular (CV) risk factors is poorly understood. We profiled a range of circulating endothelial function, oxidative stress and inflammatory biomarkers and explored their associations with individual CV risk factors in young (20–30 years, *n* = 1196) adults without self-reported chronic conditions or medication use. Participants were stratified into CV risk factor groups based on blood pressure, anthropometrical measurements, biochemical analyses and questionnaire data. We identified several differences in biomarker levels between control (without any CV risk factors) and individual CV risk factor groups and confirmed independent associations of these biomarkers with individual risk factors (all *p* < 0.05). Greater central adiposity was mainly associated with a pro-inflammatory profile (interleukin-6, C-reactive protein and fibrinogen), but also with endothelial activation and oxidative stress (plasminogen activator inhibitor-1 and reactive oxygen species). High low-density lipoprotein cholesterol levels and alcohol use were associated with endothelial activation (P-selectin), while smoking, low high-density lipoprotein cholesterol levels and alcohol use were related to markers of inflammation (growth differentiation factor-15 and monocyte chemoattractant protein-1). Glycated hemoglobin levels and blood pressure were positively associated with glutathione reductase activity, while blood pressure also associated positively with interleukin-10 levels. Our data provides insight into the initial underlying mechanisms associated with early CVD development in young individuals exposed to different cardiovascular risk factors.

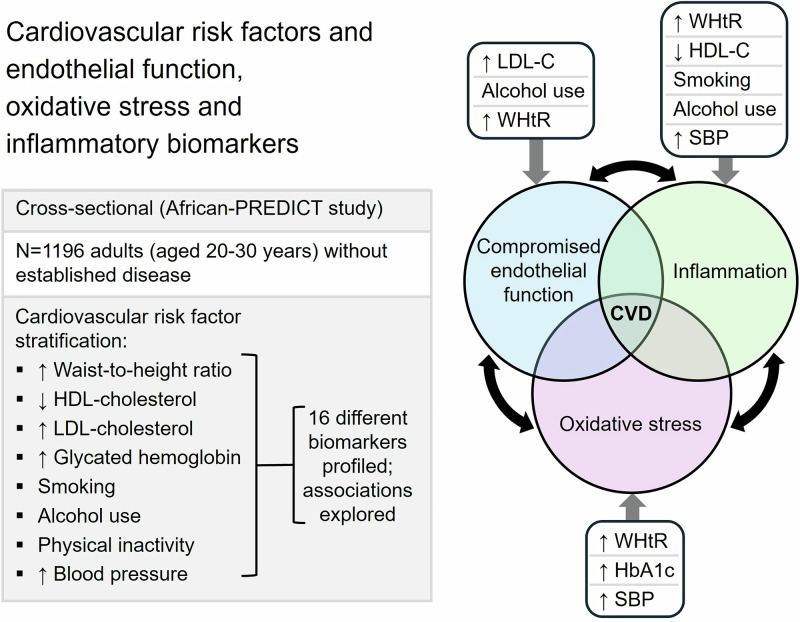

## Introduction

The growing prevalence of cardiovascular disease (CVD) in children and young adults has been highlighted in recent years [1, 2]. Several risk factors, most of which are modifiable, are known to contribute to the development of CVD. These risk factors include obesity, high blood pressure, physical inactivity, hyperglycaemia, dyslipidaemia, smoking [3], and excessive alcohol consumption [4]. Some of these risk factors, either individually or combined, may already be present in childhood [5] and have been shown to adversely affect cardiovascular health from a young age, tracking into adulthood [6–8].

The underlying processes by which risk factors contribute to the early development of CVD [9] include endothelial activation or impaired endothelial function, increasing oxidative stress and pro-inflammation, which are reflected by changes in the levels of specific biomarkers [10–12]. The differential activation of these pathways in response to different risk factors is not fully understood. Although previous studies have investigated the link between risk factors and circulating biomarkers [13, 14], these were generally in older individuals, or limited biomarkers and risk factors being investigated in the absence of a no-risk control group. Thus, there is a need to explore more comprehensive biomarker profiles – specifically in young individuals and in the absence of diagnosed CVD, to unravel the interaction of these biological pathways with CV risk factors to further gain insight into the early stages of CVD development. The aim of this study was to profile a comprehensive range of biomarkers that are generally considered to reflect endothelial function, oxidative stress and inflammation, and for which data were available in the African-PREDICT study. These include endothelial function (soluble intercellular adhesion molecule-1 (sICAM-1), soluble vascular cell adhesion molecule-1 (sVCAM-1), P-selectin, plasminogen activator inhibitor-1 (PAI-1), and von Willebrand factor (vWF)), oxidative stress (total glutathione (tGSH), glutathione reductase (GR) activity, superoxide dismutase (SOD) activity, glutathione peroxidase (GPx) activity, and reactive oxygen species (ROS)), and inflammatory (interleukin-10 (IL-10), interleukin-6 (IL-6), C-reactive protein (CRP), fibrinogen, monocyte chemoattractant protein-1 (MCP-1), and growth differentiation factor-15 (GDF-15)) biomarkers in participants with varying cardiovascular risk factors in a young South African adult cohort.

## Materials and Methods

### Study population and protocol

This study is embedded in the African Prospective study on the Early Detection and Identification of Cardiovascular disease and Hypertension (African-PREDICT) [15]. In summary, this study employs a longitudinal design to explore the early development of cardiovascular disease-related mechanisms by tracking young (20-30 years old) healthy Black and White adults over time. Prior to being included in the African-PREDICT study, participants underwent a screening process to determine their eligibility. Individuals were eligible for inclusion if they had normal office blood pressure ( < 140/90 mmHg), were HIV-uninfected, had no diagnosed chronic illnesses or reported taking medication for such conditions, and were not pregnant or breastfeeding. The African-PREDICT study adheres to all relevant ethical standards outlined in the Declaration of Helsinki for research involving human participants and is registered on ClinicalTrials.gov (NCT03292094). Participants provided written informed consent, and the Health Research Ethics Committee of the North-West University granted approval for the study (NWU-00001-12-A1). Baseline data were collected from *n* = 1202 participants, of which *n* = 4 participants were excluded from the current study due to the use of anti-inflammatory medication and *n* = 2 were excluded due to missing data for the biomarkers of interest, resulting in a total of *n* = 1196 participants.

### Questionnaire data

Comprehensive health and demographic questionnaires were completed to gather data on age, sex, ethnicity, medication use, smoking and alcohol consumption, education level, employment information and household income. Socio-economic status (SES) was determined using the Kuppuswamy’s Socioeconomic Status Scale point system adapted for the South African environment [16]. Participants received scores in three distinct categories namely i) skill level based on the South African Standard Classification of Occupation, ii) education, and iii) income levels. These elements were used to calculate participants’ SES scores.

The Global Physical Activity Questionnaire (GPAQ) was used to gather physical activity data [17, 18]. The collected data included information about sedentary behaviour as well as moderate and vigorous intensity physical activity during work, transport and recreational activities. Metabolic equivalents (METs) were calculated, where one MET corresponds to the energy expended during quiet sitting and is equivalent to a caloric expenditure of 1 kCal/kg/hour. Moderate-intensity physical activities were assigned 4 METs, while vigorous-intensity physical activities were assigned 8 METs.

### Anthropometric measurements

Anthropometric measurements were conducted following the protocols outlined by the International Society for the Advancement of Kinanthropometry (ISAK) [19]. These measurements included height (cm) (SECA 213 portable stadiometer, Hamburg, Germany), and waist circumference (cm) (Lufkin steel anthropometric tape, Apex, USA), whereafter the waist-to-height ratio (WHtR) was calculated.

### Blood pressure measurements

The Dinamap Procare 100 Vital Signs Monitor (GE Medical Systems, Milwaukee, USA) with appropriately sized cuffs were used to measure office blood pressure. Participants were instructed not to smoke, exercise, or eat prior to conducting the measurements, which were taken in a seated, relaxed position with the participant’s arm supported at heart level. The initial measurement was taken on the left arm after the participant was seated for at least 5 minutes. Subsequently, blood pressure was measured in duplicate on the right arm followed by a final measurement on the left arm. For each measurement brachial systolic blood pressure (bSBP), diastolic blood pressure (bDBP), and heart rate were recorded. The mean of the left and right arm measurements was used in data analysis.

### Biochemical analyses

Fasting blood samples were collected, prepared, aliquoted and stored at –80 °C until analysis. High-density lipoprotein cholesterol (HDL-C, mmol/L), low-density lipoprotein cholesterol (LDL-C, mmol/L), and CRP (high-sensitivity, mg/L) were measured in serum samples, and glycated haemoglobin (HbA1c, %) was measured in EDTA whole blood samples, using the Cobas Integra 400plus (Roche, Basel, Switzerland). The Quantikine ELISA kit (R&D systems, Minneapolis, MN, USA) was used for measuring IL-6 (high-sensitivity, pg/mL), sICAM-1 (ng/mL), and sVCAM-1 (ng/mL) in serum samples, and MCP-1 (pg/mL) in EDTA plasma samples, on a Synergy H4 hybrid microplate reader (BioTek, Winooski, VT, USA).

Serum GR (U/L) (Randox, Crumlin, UK), EDTA whole blood SOD (U/mL) (Randox, Crumlin, UK) and GPx (U/L) (Randox; Crumlin, UK) were also measured on the Cobas Integra 400 plus (Roche, Basel, Switzerland). Serum ROS (Units [where 1 unit = 1.0 mg/L H_2_O_2_]) were measured using a high-throughput spectrophotometric assay [20] and EDTA whole blood tGSH (µM) was measured using a Biotech® GSH/GSSG-412™ assay (OxisResearch; CA; USA) on a Synergy H4 hybrid microplate reader (BioTek, Winooski, VT, USA).

Serum GDF-15 (ng/mL), P-selectin (ng/mL) (Milliplex Map Human Cardiovascular Disease Magnetic Bead Panel 2, Merck Millipore, Darmstadt, Germany), and IL-10 (pg/mL) (Human High-Sensitivity T Cell Magnetic Bead Panel 96-Well Plate Assay) were measured using a Luminex 200^TM^ analyser (Luminex, Austin, TX, US), and serum cotinine (ng/mL) was measured using a chemiluminescence method on the Immulite system (Siemens, Erlangen, Germany).

Citrated plasma samples were used for measuring fibrinogen (g/L) (with a modified Clauss method on the ACL-200 (Instrumentation Laboratories, Milan, Italy)), PAI-1 activity (PAI-1_act_, IU/mL) (with the Technozym^®^ PAI-1 Actibind^®^ ELISA kit (Technoclone, Vienna, Austria)) and vWF antigen (vWF_ag_, %) levels (using a sandwich enzyme-linked immunosorbent assay). The latter was performed using polyclonal rabbit anti-vWF antibody and rabbit anti-vWF horseradish peroxidase (HRP) antibody (DAKO, Glostrup, Denmark), and the standard curve for quantification was created using the 6^th^ International Standard for vWF/FVIII.

### Classification of the control and risk factor groups

Risk groups based on the risk factor data were categorized as follows. The WHtR was used to classify participants into overweight/obese (risk group) (WHtR ≥ 0.55) and normal weight (no risk group) (WHtR < 0.55) [21, 22]. The South African Heart Association (SA Heart) and the Lipid and Atherosclerosis Society of Southern Africa (LASSA) guidelines were used for the classification of HDL-C and LDL-C risk groups [23]. The HDL-C risk groups were categorized based on HDL-C ≤ 1.0 mmol/L (men) and HDL-C ≤ 1.2 mmol/L (women), while the no-risk groups were categorized based on HDL-C > 1.0 mmol/L (men) and HDL-C > 1.2 mmol/L (women). For LDL-C, the risk group was defined based on LDL-C ≥ 3.0 mmol/L, and the no-risk group as LDL-C < 3.0 mmol/L [23]. Participants were classified as prediabetic/diabetic (risk group) based on HbA1c levels ≥ 5.7%, and as the no-risk group if HbA1c levels were less than 5.7% [24]. Participants who indicated that they smoked or had serum cotinine levels ≥11 ng/mL were classified as the smoking risk group, whereas those who indicated that they were non-smokers and had cotinine levels <11 ng/mL were classified as the no-risk group [25, 26]. Alcohol use based on the questionnaire data (yes/no) was used to classify the alcohol risk and no-risk groups. The combination of moderate- and vigorous physical activity MET-minutes/week was used to stratify participants into two categories: i) active (no risk group) ( ≥ 600 MET-minutes/week of combined moderate- and vigorous-intensity physical activity) and ii) inactive (risk group) ( < 600 MET-minutes/week of combined moderate- and vigorous-intensity physical activity) [18]. Blood pressure classification was based on the 2020 International Society of Hypertension guidelines [27] where bSBP<130 mmHg and bDBP<85 mmHg was considered normotensive (no risk group) and bSBP ≥130 mmHg and/or bDBP ≥85 mmHg as pre-hypertensive or hypertensive (risk group). The control group for the study was comprised of participants who had none of the risk factors as specified above.

### Statistical analysis

IBM^®^ SPSS^®^ Statistics version 29 software (IBM Corporation; Armonk, New York, USA) was used for statistical analyses and GraphPad Prism version 5.03 (GraphPad Software Inc., CA, USA) for the graphical representation of data. Continuous data were inspected for normality using Q-Q plots and skewed data were logarithmically transformed. Log-transformed variables included WC, WHtR, total moderate and vigorous physical activity per week, cotinine, IL-10, IL-6, CRP, PAI-1_act_, sVCAM-1, P-selectin, MCP-1, GDF-15, vWF, and ROS. Data were reported as arithmetic means and standard deviations, geometric means with 5^th^ and 95^th^ percentile intervals, and counts and proportions for normally distributed, log-transformed, and categorical data, respectively. Biomarker levels were compared between the control and risk groups using analysis of covariance (ANCOVAs). The latter were adjusted for age, sex, ethnicity, and SES score. Cardiovascular risk factors are known to be interrelated and, since we aimed to investigate individual risk factor groups, we statistically accounted for potential confounding by additionally adjusting for all other risk factors (continuous variables [with the exception of smoking and alcohol where the categorical variables were used]). Biomarkers that differed significantly between the control and risk groups were further explored in partial correlations between the identified biomarkers and risk factors, adjusted for age, sex, and ethnicity. This was followed by backward multiple regression analysis in the total group and the respective risk groups with the identified biomarkers as dependent variables. All risk factors (continuous variables, except for smoking and alcohol [categorical variables]) as well as age, sex, ethnicity, and SES score, were included as independent variables. Continuous variables were normalised (z-transformed) for the multiple regression analyses. A *p*-value < 0.05 was regarded as statistically significant.

## Results

**Fig. 1:**
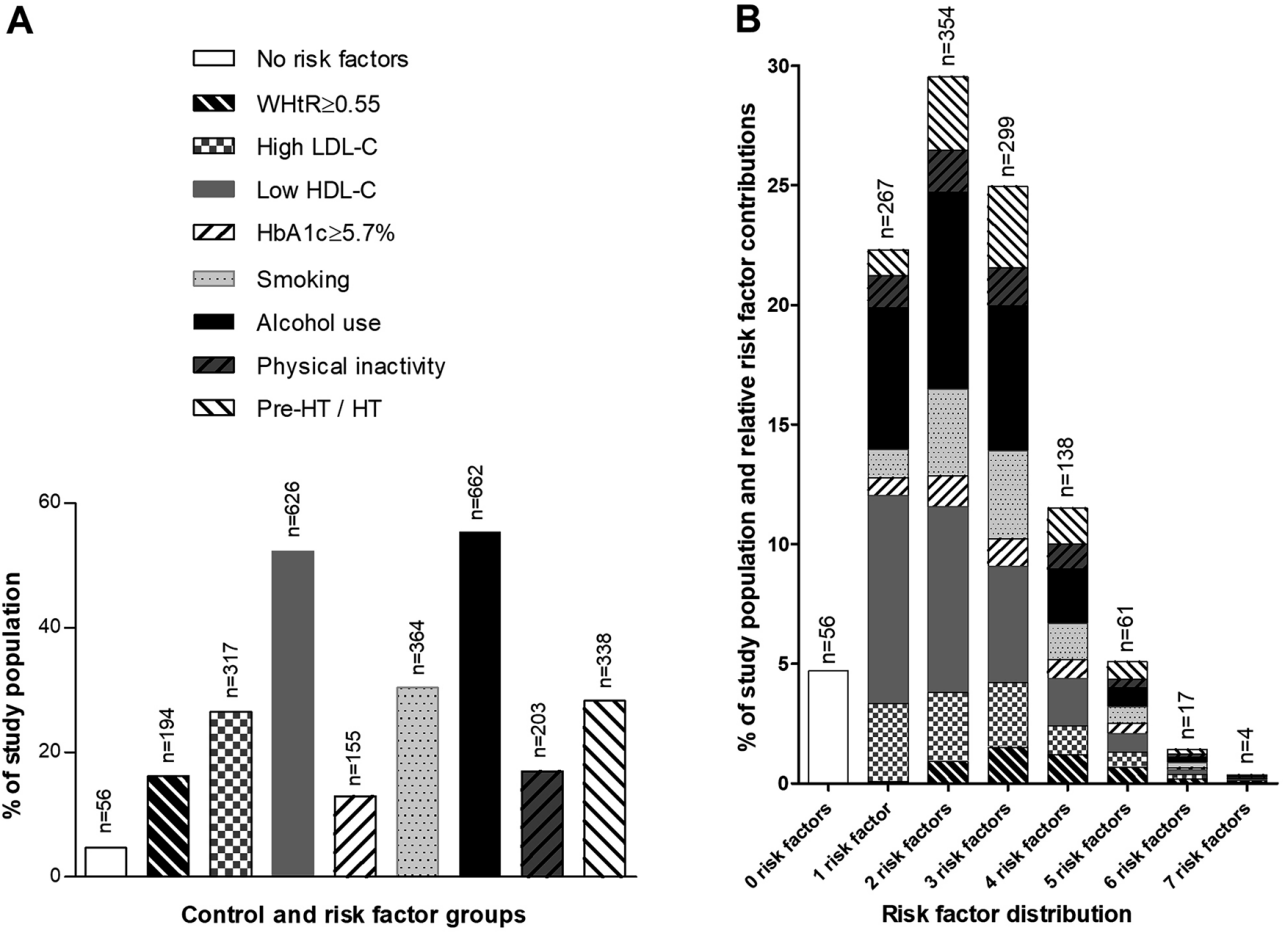
Distribution of control and risk factors in the study population (**A**) and within participants exposed to one or more risk factors (**B**). Abbreviations: WHtR, weight-to-height ratio; LDL-C, low-density lipoprotein cholesterol; HDL-C, high-density lipoprotein cholesterol; HbA1c, glycated haemoglobin; Pre-HT, prehypertension; HT, hypertension

The general characteristics of the study population are shown in Table 1. Participants had a mean age of 24.5 ± 3.12 years and were equally distributed in terms of sex (48.2% men vs 51.8% women) and ethnicity (50.4% Black vs 49.6% White). The distribution of the control and respective risk factor groups is depicted in Fig. 1, showing low HDL-C levels and alcohol use as the two most prominent cardiovascular risk factors in this cohort.

**Table 1:** Characteristics of the study population (*n* = 1196)

Age (years)	24.5 ± 3.12
Sex (men), n (%)	576 (48.2)
Ethnicity (Black), n (%)	604 (50.5)
Socio-economic status score	20.6 ± 6.12
***Anthropometric***	
Waist circumference (cm)	79.2 (63.9; 103)
Body height (cm)	168 ± 9.52
Waist-to-height ratio	0.47 (0.39; 0.61)
***Lifestyle***	
Self-reported smoking (yes), n (%)	285 (23.8)
Self-reported alcohol consumption* (yes), n (%)	662 (55.7)
Total moderate and vigorous physical activity per week (MET-minutes)	2604 (320; 17628)
***Cardiovascular***	
Office systolic blood pressure (mmHg)	119 ± 11.7
Office diastolic blood pressure (mmHg)	78.6 ± 7.66
***Biochemical***	
High-density lipoprotein cholesterol (mmol/L)	1.16 ± 0.42
Low-density lipoprotein cholesterol (mmol/L)	2.44 ± 0.98
Glycated haemoglobin (%)	5.32 ± 0.31
Cotinine (ng/mL)	3.59 (1.00; 322)
*Endothelial function markers*	
Soluble intercellular adhesion molecule-1 (ng/mL)	181 ± 75.2
Soluble vascular cell adhesion molecule-1 (ng/mL)	627 (394; 986)
P-selectin (ng/mL)	117 (70.3; 195)
Plasminogen activator inhibitor-1 activity (IU/mL)	5.02 (0.25; 41.2)
Von Willebrand factor (%)	84.0 (41.0; 185)
*Oxidative stress markers*	
Total glutathione (µM)	781 ± 294
Glutathione reductase (U/L)	39.6 ± 17.5
Superoxide dismutase (U/mL)	4.48 ± 1.90
Glutathione peroxidase (U/L)	9697 ± 2773
Reactive oxygen species (Units)^1^	38.6 (12.7; 108)
*Inflammatory markers*	
Interleukin-10 (pg/mL)	4.89 (1.01; 21.2)
Interleukin-6 (pg/mL)	1.08 (0.38; 3.76)
C-reactive protein (mg/L)	0.89 (0.08; 9.52)
Fibrinogen (g/L)	2.50 ± 0.59
Monocyte chemoattractant protein-1 (pg/mL)	148 (94.2; 238)
Growth differentiation factor-15 (ng/mL)	0.33 (0.19; 0.59)

Data reported as arithmetic means ± standard deviations, geometric means with 5^th^ and 95^th^ percentiles, or counts and percentages

*Data available for *n* = 1188 participants

^1^Reactive oxygen species measured as serum peroxides where 1 unit=1.0 mg/L H_2_O_2_

Biomarker levels were first compared between the control group and each of the risk groups. After adjusting for age, sex, ethnicity, SES score, and all other investigated risk factors (Table 2), P-selectin levels were higher in the LDL-C and alcohol risk groups (*p* ≤ 0.043), and PAI-1_act_ levels were higher in die WHtR risk group (*p* = 0.001) but lower in the alcohol risk group (*p* = 0.042) compared to the control group. The oxidative stress profile showed higher levels of GR in the WHtR, HbA1c, physical inactivity, and blood pressure risk groups, as well as higher levels of ROS in the WHtR risk group compared to the control group (all *p* ≤ 0.035). Among the inflammatory markers, IL-6, CRP, and fibrinogen levels (all *p* ≤ 0.002) were higher in the WHtR risk group, and higher CRP levels were also found in the physical inactivity and blood pressure risk groups (*p* ≤ 0.034). GDF-15 levels were higher in the smoking, alcohol consumption, and physical activity risk groups, MCP-1 was higher in the HDL-C risk group, and IL-10 was lower in the smoking and blood pressure risk groups compared to the control group (all *p* ≤ 0.044).

**Table 2:** Analysis of covariance comparisons of biomarker concentrations between the control (C) and risk factor (R) groups

	Waist-to-height ratio		High-density lipoprotein cholesterol		Low-density lipoprotein cholesterol		Glycated haemoglobin		Smoking		Alcohol		Physical inactivity		Prehypertension and hypertension	
	C	R (*n* = 194)	C	R (*n* = 626)	C	R (*n* = 317)	C	R (*n* = 155)	C	R (n = 364)	C	R (n = 662)	C	R (n = 203)	C	R (n = 338)
**Endothelial function markers**																
sICAM-1 (ng/mL)	186 (156; 217)	192 (178; 206)	183 (162; 204)	189 (183; 195)	179 (158; 200)	188 (180; 196)	166 (137; 195)	174 (158; 189)	173 (148; 197)	191 (182; 199)	180 (160; 200)	178 (172; 183)	180 (159; 201)	179 (169; 189)	186 (161; 210)	178 (169; 187)
sVCAM-1 (ng/mL)	667 (600; 743)	597 (568; 627)	649 (595; 706)	631 (616; 646)	638 (583; 699)	624 (602; 647)	646 (578; 722)	610 (575; 647)	622 (564; 688)	635 (614; 656)	652 (600; 709)	614 (600; 629)	628 (575; 685)	637 (612; 664)	619 (566; 680)	624 (604; 645)
P-selectin (ng/mL)	112 (100; 125)	119 (114; 126)	116 (106; 126)	114 (111; 117)	114 (103; 125)	126 (122; 131)*	109 (98; 121)	110 (104; 116)	116 (105; 128)	114 (110; 118)	110 (101; 119)	121 (118; 124)*	111 (102; 122)	120 (115; 125)	112 (101; 125)	118 (114; 123)
PAI-1_act_ (IU/mL)	5.46 (3.74; 7.98)	12.0 (9.99; 14.3)**	7.52 (5.28; 10.7)	5.24 (4.73; 5.80)	7.48 (5.20; 10.7)	5.89 (5.10; 6.79)	6.27 (4.14; 9.50)	4.14 (3.30; 5.19)	6.24 (4.23; 9.18)	5.46 (4.78; 6.24)	7.59 (5.35; 10.8)*	5.19 (4.70; 5.73)	5.83 (3.89; 8.75)	5.26 (4.34; 6.37)	7.43 (5.01; 11.0)	5.43 (4.71; 6.26)
vWF (%)	97.5 (81.0; 117)	83.4 (76.6; 90.7)	81.8 (72.6; 92.4)	86.1 (83.2; 89.1)	78.3 (68.0; 90.5)	84.3 (79.7; 89.3)	83.9 (70.4; 100)	93.3 (85.1; 102)	90.6 (79.2; 104)	80.9 (77.3; 84.7)	83.9 (74.6; 94.4)	83.0 (80.2; 85.7)	84.3 (72.4; 98.1)	82.4 (76.8; 88.4)	87.1 (75.3; 101)	83.0 (78.9; 87.4)
**Oxidative stress markers**																
tGSH (µM)	755 (645; 865)	782 (732; 832)	768 (694; 842)	718 (697; 739)	772 (688; 856)	835 (802; 869)	737 (620; 854)	875 (813; 937)	807 (710; 905)	814 (781; 847)	782 (698; 866)	812 (788; 836)	766 (681; 852)	778 (738; 818)	778 (684; 872)	787 (753; 820)
GR (U/L)	33.9 (26.5; 41.4)	44.8 (41.3; 48.2)^*^	35.1 (30.7; 39.6)	37.2 (35.9; 38.5)	37.3 (31.9; 42.7)	42.2 (10.0; 44.4)	35.0 (29.2; 40.8)	44.2 (41.1; 47.2)^*^	36.3 (30.8; 41.9)	38.2 (36.4; 40.1)	35.9 (31.1; 40.6)	40.3 (39.0; 41.7)	33.1 (27.7; 38.4)	41.6 (39.1; 44.2)^**^	34.7 (28.7; 40.6)	42.8 (40.6; 44.9)^*^
SOD (U/mL)	4.69 (3.83; 5.56)	4.65 (4.26; 5.05)	4.81 (4.31; 5.31)	4.81 (4.67; 4.95)	4.61 (3.98; 5.23)	4.06 (3.81; 4.31)	4.99 (4.28; 5.70)	4.29 (3.91; 4.66)	4.73 (4.11; 5.35)	4.44 (4.23; 4.65)	4.82 (4.29; 5.34)	4.29 (4.15; 4.44)	4.47 (3.88; 5.06)	4.52 (4.24; 4.79)	4.55 (3.92; 5.18)	4.43 (4.20; 4.65)
GPx (U/L)	9051 (8001; 10101)	9267 (8787; 9748)	9484 (8684; 10284)	9754 (9527; 9982)	9312 (8487; 10137)	9221 (8895; 9548)	9064 (8008; 10119)	9867 (9306; 10427)	9197 (8321; 10073)	9759 (9462; 10057)	9207 (8441; 9973)	9929 (9711; 10147)	9287 (8424; 10151)	9427 (9023; 9831)	9694 (8767; 10621)	9731 (9400; 10061)
ROS (Units)^1^	35.2 (27.7; 44.6)	49.8 (44.6; 55.5)^*^	38.6 (32.6; 45.8)	35.2 (33.5; 36.9)	38.8 (32.1; 46.9)	41.8 (38.7; 45.0)	36.5 (29.8; 44.6)	46.3 (41.7; 51.6)	37.2 (30.5; 45.5)	38.3 (35.8; 41.0)	36.1 (30.6; 42.4)	39.8 (38.0; 41.7)	38.6 (31.5; 47.4)	40.7 (37.0; 44.9)	33.0 (27.4; 39.9)	40.5 (37.8; 43.2)
**Inflammatory markers**																
IL-10 (pg/mL)	5.41 (3.74; 7.80)	4.30 (3.64; 5.07)	5.64 (4.30; 7.39)	4.98 (4.61; 5.38)	5.81 (4.38; 7.71)	4.89 (4.38; 5.47)	5.70 (4.06; 8.02)	4.45 (3.72; 5.32)	6.61 (4.81; 9.06)*	4.53 (4.06; 5.04)	6.28 (4.81; 8.20)	4.79 (4.43; 5.16)	5.14 (3.81; 6.93)	5.04 (4.38; 5.78)	6.44 (4.73; 8.78)*	4.46 (4.00; 4.97)
IL-6 (pg/mL)	1.04 (0.84; 1.29)	1.69 (1.53; 1.87)***	1.08 (0.91; 1.28)	1.26 (1.20; 1.32)	1.10 (0.93; 1.31)	0.95 (0.89; 1.02)	1.12 (0.92; 1.35)	1.28 (1.16; 1.41)	1.02 (0.83; 1.26)	1.10 (1.02; 1.18)	1.14 (0.97; 1.34)	1.00 (0.95; 1.05)	1.03 (0.85; 1.25)	1.15 (1.05; 1.25)	1.07 (0.88; 1.30)	1.10 (1.02; 1.18)
CRP (mg/L)	0.56 (0.38; 0.83)	3.00 (2.50; 3.59)***	0.69 (0.49; 0.98)	0.94 (0.85; 1.04)	0.86 (0.59; 1.23)	1.16 (1.01; 1.34)	0.83 (0.54; 1.27)	1.42 (1.13; 1.78)	0.70 (0.47; 1.05)	0.76 (0.66; 0.88)	0.69 (0.49; 0.97)	0.85 (0.77; 0.93)	0.72 (0.49; 1.06)	1.24 (1.03; 1.48)*	0.58 (0.39; 0.86)	0.94 (0.81; 1.08)*
Fibrinogen (g/L)	2.48 (2.26; 2.69)	2.89 (2.79; 2.99)**	2.43 (2.29; 2.58)	2.55 (2.51; 2.59)	2.42 (2.25; 2.59)	2.59 (2.53; 2.66)	2.53 (2.35; 2.70)	2.75 (2.65; 2.84)	2.37 (2.21; 2.54)	2.40 (2.35; 2.46)	2.44 (2.30; 2.59)	2.46 (2.42; 2.50)	2.41 (2.24; 2.58)	2.58 (2.50; 2.66)	2.42 (2.25; 2.58)	2.44 (2.38; 2.50)
MCP-1 (pg/mL)	139 (125; 154)	151 (144; 158)	139 (129; 149)	154 (151; 157)*	139 (129; 151)	141 (137; 146)	145 (129; 162)	162 (153; 172)	145 (133; 157)	153 (149; 157)	144 (134; 155)	147 (144; 150)	141 (131; 151)	140 (135; 145)	149 (135; 164)	158 (153; 164)
GDF-15 (ng/mL)	0.32 (0.28; 0.36)	0.33 (0.31; 0.35)	0.30 (0.28; 0.34)	0.32 (0.31; 0.33)	0.32 (0.28; 0.35)	0.34 (0.33; 0.36)	0.31 (0.27; 0.35)	0.35 (0.33; 0.38)	0.31 (0.27; 0.35)	0.35 (0.34; 0.37)*	0.30 (0.28; 0.33)	0.34 (0.33; 0.35)*	0.31 (0.28; 0.34)	0.35 (0.33; 0.36)*	0.31 (0.27; 0.34)	0.33 (0.32; 0.35)

Adjusted for age, sex, ethnicity, SES score, and all other risk factors (continuous variables [with the exception of smoking and alcohol where the categorical variables were used])

Reported as estimated means and 95% confidence intervals

Significance level: *p < 0.05; **p < 0.01; ***p < 0.001

^1^Reactive oxygen species measured as serum peroxides where 1 unit=1.0 mg/L H_2_O_2_

Abbreviations: sICAM-1, soluble intercellular adhesion molecule-1; sVCAM-1, soluble vascular cell adhesion molecule-1; PAI-1_act_, plasminogen activator inhibitor-1 activity; vWF, von Willebrand factor; tGSH, total glutathione; GR, glutathione reductase; SOD, superoxide dismutase; GPx, glutathione peroxidase; ROS, reactive oxygen species; IL-10, interleukin-10; IL-6, interleukin-6; CRP, C-reactive protein; MCP-1, monocyte chemoattractant protein-1; GDF-15, growth differentiation factor-15

**Fig. 2:**
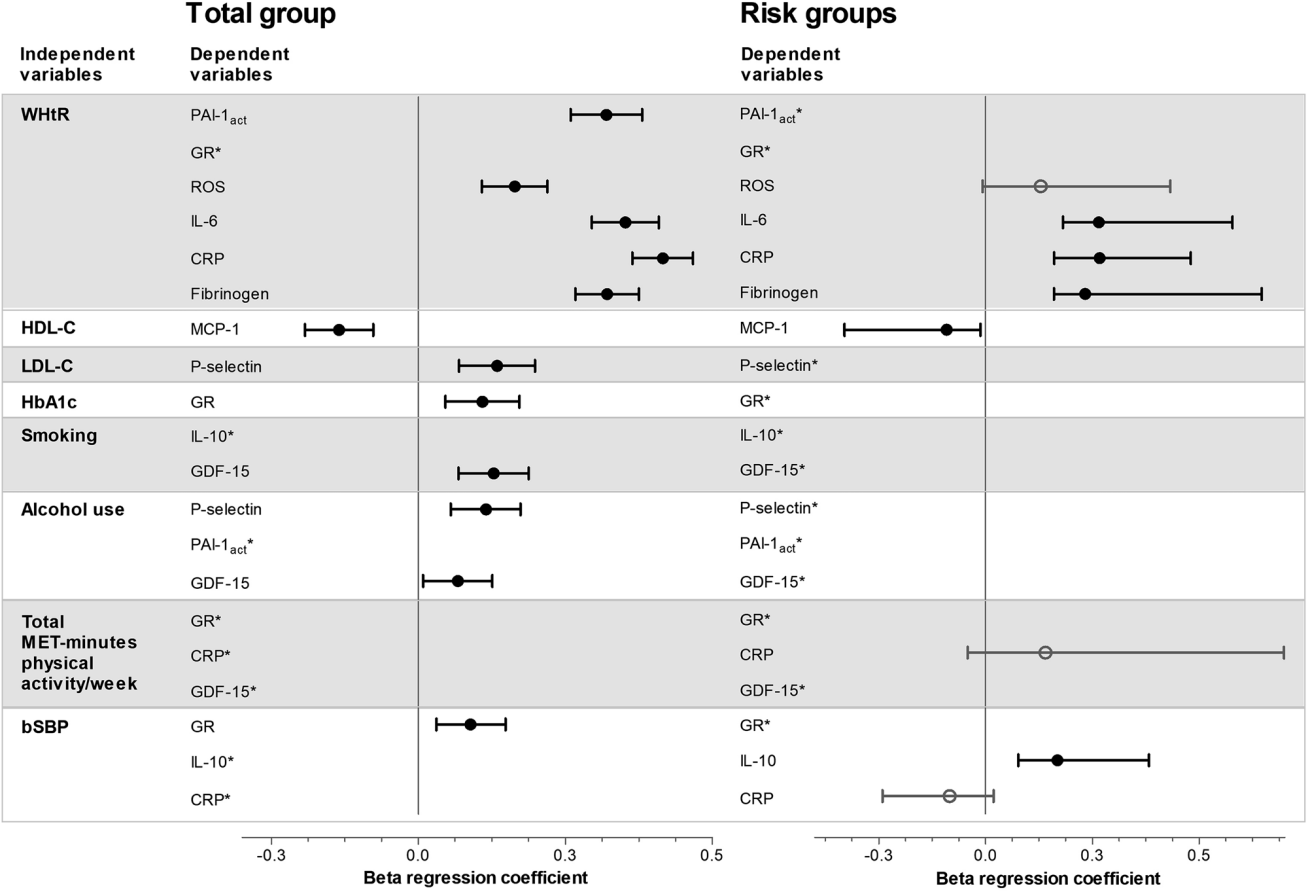
Backward multiple regression analyses with selected biomarkers as dependent variables and risk factors as independent variables in the total group and the respective risk groups, presented as standardized β and 95% CIs. Independent variables included in all models: age, sex, ethnicity, SES score, WHtR, HDL-C, LDL-C, HbA1c, smoking (categorical) alcohol (categorial), total MET-minutes physical activity/week, and bSBP. *Main independent variable did not contribute to the final regression model. Abbreviations: WHtR, waist-to-height ratio; PAI-1_act_, plasminogen activator inhibitor-1 activity; GR, glutathione reductase; ROS, reactive oxygen species; IL-6, interleukin-6; CRP, C-reactive protein; HDL-C, high-density lipoprotein cholesterol; MCP-1, monocyte chemoattractant protein-1; LDL-C, low-density lipoprotein cholesterol; HbA1c, glycated haemoglobin; IL-10, interleukin-10; GDF-15, growth differentiation factor-15; bSBP, brachial systolic blood pressure

Biomarkers that differed between the control and risk factor groups based on the ANCOVA results were further investigated in the total group using partial correlations adjusted for age, sex, and ethnicity (Supplementary Table 1), followed by confirmation by multiple regression analysis. Physical activity correlated negatively and bSBP positively with CRP in the total group using partial correlations (*p* ≤ 0.036), but neither were significant in multiple regression analysis. All other associations found using partial correlations were confirmed in multiple regression analyses (Fig. 2, Supplementary Tables 2A-H).

For the endothelial function markers in the total group, P-selectin was positively associated with LDL-C (β = 0.13, *p* < 0.001) and alcohol consumption (β = 0.12, *p* < 0.001), and PAI-1_act_ was positively associated with WHtR (β = 0.32, *p* < 0.001). For the oxidative stress markers, GR was positively associated with HbA1c (β = 0.11, *p* = 0.001) and bSBP (β = 0.09, *p* = 0.003), and ROS was positively associated with WHtR (β = 0.16, *p* < 0.001). None of these associations were significant in the respective risk groups.

Several inflammatory markers were associated with risk factors in the total group and the respective risk groups. IL-6, CRP, and fibrinogen associated positively with WHtR in both the total group and within the WHtR risk group: IL-6 (β = 0.35, *p* < 0.001 in the total group; β = 0.27, *p* < 0.001 in the WHtR risk group), CRP (β = 0.42, *p* < 0.001 in the total group; β = 0.27, *p* < 0.001 in the WHtR risk group), and fibrinogen (β = 0.32, *p* < 0.001 in the total group; β = 0.23, *p* = 0.001 in the WHtR risk group). MCP-1 associated negatively with HDL-C in both the total group (β = −0.14, *p* < 0.001) and the HDL-C risk group (β = −0.09, *p* = 0.035). GDF-15 had a positive association with smoking (β = 0.13, *p* < 0.001) and alcohol consumption (β = 0.07, *p* = 0.027) in the total group only, and IL-10 associated negatively with bSBP (β = 0.17, *p* = 0.003) in the BP risk group only.

Other covariates that differentially contributed to the variance in biomarker levels in the total and risk factor groups included age, sex, ethnicity, SES score, WHtR, HDL-C, LDL-C, HbA1c, smoking, alcohol use, total MET-minutes physical activity/week, and bSBP (Supplementary Tables 2A-H).

## Discussion

The study aimed to profile various endothelial function, oxidative stress, and inflammatory biomarkers in individuals with various cardiovascular risk factors, in comparison to those without any risk factors present, in a young cohort without overt CVD. By stratifying our population by individual CV risk factors and adjusting for the alternative risk factors, we sought to gain a more comprehensive understanding of these interactions. We demonstrated that levels of certain biomarkers differ, albeit within normal ranges for the markers with clinical reference ranges, between the control and cardiovascular risk factor groups. The levels of most biomarkers were higher in the risk groups, with the inverse being the case for PAI-1_act_ in the alcohol risk group, and IL-10 in the smoking and blood pressure risk groups. Additionally, independent associations of several biomarkers with the individual risk factors (greater central adiposity, smoking, alcohol use, physical inactivity, low HDL-C and high LDL-C, HbA1c, and bSBP) were also shown. Our data highlight the link between various individual modifiable cardiovascular risk factors and circulating markers associated with CVD in a young population without established CVD, which gives insight into the initial stages of CVD development driven by exposure to specific risk factors.

Obesity is one of the most concerning risk factors globally, and its prevalence is on the increase, reaching more than 1 billion people in 2022 [28]. In South Africa, more than 50% of adults are overweight (25.3%) or obese (32.1%) [29]. In our study, we found higher PAI-1_act_, glutathione reductase, ROS, IL-6, CRP, and fibrinogen levels in the high central adiposity group compared to the control group. A recent study showed increased PAI-1_act_ and longer clot lysis time in Black South Africans with metabolic syndrome than in individuals without metabolic syndrome, and that central obesity is a main predictor of PAI-1_act_ levels [30]. The positive association between PAI-1_act_ (a marker of endothelial dysfunction) and WHtR in our study is likely largely due to adipose tissue, especially visceral adipose tissue, being a major source of PAI-1 production [31], although obesity is also known to involve endothelial dysfunction [32]. Obesity is also closely linked to increased ROS, oxidative stress [33], and inflammation [34], which is consistent with our findings. Chronic low-grade inflammation is a well-known hallmark of excess body fat [34], and in our study we also confirmed positive associations of the inflammatory markers IL-6, CRP, and fibrinogen with central adiposity in both the total and high WHtR groups. These findings suggest that young adults with a higher degree of central adiposity may be at risk of compromised endothelial function, impaired redox regulation and chronic pro-inflammation.

The prevalence of early-onset atherosclerotic CVD is on the increase [35], with the development of early atherosclerotic signs already occurring in childhood and young adulthood. The extent of atherosclerotic lesions has been related to various cardiovascular risk factors, including elevated LDL-C and lower HDL-C to low-density plus very-low-density lipoprotein cholesterol ratio [36, 37]. The Coronary Artery Risk Development in Young Adults (CARDIA) study conducted on young adults (aged 18-30 years) emphasized the importance of optimal LDL-C levels early in life, as incident CVD event risk increased with cumulative LDL-C exposure after a median follow-up of 16 years [38]. We found that individuals with high LDL-C and low HDL-C levels had augmented levels of circulating P-selectin and MCP-1, respectively, independent of other cardiovascular risk factors. These differences were supported by independent associations between the respective biomarkers and lipid fractions in the total population, and for MCP-1 in the HDL-C risk group. Both findings are in accordance with previous studies, albeit in adults of an older age or where other co-morbidities were present [39, 40]. LDL-C, upon oxidation, is thought to stimulate the expression of P-selectin in endothelial cells [41], and LDL-apheresis has been shown to reduce plasma P-selectin levels [42]. In line with the risk that high LDL-C levels pose for atherosclerosis, P-selectin was shown to be positively associated with aortic wall thickness in young adults (aged 25–35 years) from the general population, which the authors suggested may indicate atherogenic inflammatory changes in the arterial wall in young adulthood [43]. With regards to HDL-C, experimental studies have shown that HDL-C is able to inhibit endothelial MCP-1 secretion, a process dependent on annexin A1 upregulation [44]. Mechanistically, this would support the inverse association found in our study. Given that both P-selectin and MCP-1 have predictive value for cardiovascular events and mortality, respectively [45, 46], our data contribute to the understanding of the preclinical biochemical changes that may occur with early cardiovascular risk development in the setting of high LDL-C or low HDL-C levels in young adults.

In our study, we observed higher GR levels in the HbA1c and blood pressure risk groups compared to the control group, and GR levels showed positive associations with HbA1c and bSBP in the total cohort. Diabetes is regarded as a classical risk factor for CVD. However, even in the general population without diabetes, elevated HbA1c levels were found to be associated with CVD incidence and cardiovascular mortality [47]. Oxidative stress is considered to be a central mechanism that links diabetes to CVD, and it contributes to both conditions through the overproduction of ROS, with subsequent cellular and vascular damage [48]. The ability to combat oxidative stress and its resulting damage depends on the efficiency of various antioxidant defence mechanisms, including the glutathione cycle [49]. Previous studies have highlighted glutathione deficiency in diabetic individuals [50, 51], which is attributed to both increased utilization and impaired synthesis of glutathione, where GR plays a key role in regenerating the reduced form of glutathione (GSH) from its oxidized counterpart (GSSG) [49]. While the associations of GR with HbA1c and bSBP were evident across the total study population, our findings suggest that GR activity is likely upregulated in prediabetic/diabetic and prehypertensive/hypertensive individuals to support increased glutathione turnover and maintain the balance between GSSG and GSH. We also found lower levels of the anti-inflammatory marker IL-10 in the blood pressure risk group compared to the control group, and a positive association between IL-10 and bSBP only in the blood pressure risk group. Previous research has highlighted the protective role of IL-10 in preserving vascular function [52], which aligns with our finding of reduced IL-10 levels under prehypertensive/hypertensive conditions. However, the observed positive association between IL-10 and bSBP only in the blood pressure risk group might represent a paradoxical compensatory anti-inflammatory response to counteract inflammation associated with high blood pressure.

We found higher GDF-15 levels in the smoking and alcohol consumption risk groups compared to the control group, as well as positive associations of GDF-15 with smoking and alcohol use in the total group. This supports the findings of other studies where GDF-15 levels are associated positively with smoking [53, 54] and alcohol consumption [55]. GDF-15 is a stress-induced cytokine and is considered a marker of inflammation, oxidative stress, and cellular aging [56]. Both alcohol use [57] and smoking [58] are known to induce oxidative stress and inflammation, which may result in increased GDF-15 levels. We also found higher P-selectin levels in the alcohol risk group compared to the control group and confirmed a positive association between P-selectin and alcohol use in the total group. Our results support findings of a previous study conducted on apparently healthy adults aged 55 years and older that found higher P-selectin levels in individuals with moderate alcohol intake compared to abstainers, which was mainly driven by a positive association between P-selectin levels and wine consumption [59]. P-selectin is an adhesion molecule expressed by activated endothelial cells and platelets, and raised levels have been linked to greater atherosclerotic plaque progression [60] and increased risk for future cardiovascular events [46]. Moderate alcohol consumption is associated with a decrease in adhesion molecule levels, whereas high alcohol consumption is linked to vascular proinflammation and an upregulation in adhesion molecule expression [61]. The higher levels of P-selectin in the alcohol use group compared to the control group in our study may suggest that the alcohol users (based on yes/no questionnaire data) leaned towards a higher degree of alcohol consumption. Collectively, our results indicate that alcohol use in young adults is associated with proinflammation and endothelial activation.

It is well known that the underlying mechanisms that lead to CVD development and progression, i.e., impaired endothelial function, oxidative stress, and inflammation, are interconnected [62] and form a vicious cycle [10]. The initial entry point to this cycle is debatable and depends on the nature of the risk factor exposures (type, duration, alone or in combination with other risk factors). In the risk groups of our study, significant associations were only found with inflammatory markers: IL-6, CRP, and fibrinogen in the WHtR risk group, MCP-1 in the HDL-C risk group, and IL-10 only in the BP risk group, not in the total group. This collectively suggests that inflammatory pathways are the first to be activated when exposure to these specific risk factors – particularly central adiposity – exceeds beyond specific cut-off values in young adults without overt CVD.

Associations that lost statistical significance after adjusting for confounders might be attributed to mediation by other variables that were included in the models. Lack of differences in certain biomarker levels between the control and risk groups, and the absence of certain associations in our study, could be attributed to this being a young population, free of overt CVD, with biomarker levels within normal clinical reference ranges. Compensatory physiological mechanisms might still be intact in these individuals, with significant differences and associations only becoming apparent in more advanced stages of cardiovascular deterioration and risk factor exposure.

The findings of our study should be interpreted within the context of its strengths and limitations. This study was conducted on data from young individuals, which constitutes an ideal cohort for investigating the early biochemical changes associated with cardiovascular risk factor exposure. A major strength of this study is having a pure control group (without any of the risk factors investigated) for comparison with the individual risk factor groups. Our study also investigated an extensive panel of endothelial function, oxidative stress, and inflammatory biomarkers, providing a comprehensive profile of early biochemical changes related to risk factor exposure. However, we acknowledge that the control group was relatively small (*n* = 56) and, being a cross-sectional study, causal associations cannot be inferred. Participants were recruited from a defined geographical region in the North West province of South Africa and included in the study based on certain eligibility criteria, and hence our findings may not necessarily be representative of the general population. Some of the data used in our study (i.e., smoking (yes/no), alcohol consumption (yes/no) and physical activity) were derived from questionnaires, and the extent of smoking and alcohol use was not considered in this study, which might be considered a limitation. Given the interrelated nature of cardiovascular risk factors, full adjustment in ANCOVA might have attenuated some associations, potentially obscuring true effects if certain covariates act as mediators rather than confounders. Thus, the findings likely represent conservative estimates of effect sizes. Although statistical models were adjusted for potential confounders, not considering certain other factors (e.g., diet and genetics) is acknowledged as a limitation, and the possibility of residual confounding cannot be excluded. Total glutathione, constituting both GSH and GSSG, was measured in our study due to the difficulty of quantifying the low GSSG levels, which limits interpretation of our results in terms of reduced vs oxidised glutathione.

Our study showed the significance of individual modifiable cardiovascular risk factors, already at an early age, in relation to endothelial function, oxidative stress, and inflammatory profiles (albeit still within normal ranges) in young adults. Our findings also suggest that the inflammatory profile is the first to be altered in the presence of high central adiposity and blood pressure, and low HDL-C levels based on specific cut-off values. This may give insight into the initial stages of CVD development resulting from exposure to specific risk factors. It also highlights the importance of managing risk factor exposure and pursuing healthy lifestyle choices early in life to delay the development of CVD.

## Supplementary information

Supplementary Tables
