# Supplementary material for: Profiling endothelial function, oxidative stress and inflammatory biomarkers in individuals with various cardiovascular risk factors: the African-PREDICT study
Source: Hypertens Res. 2025 Oct 15;48(12):3209–20. doi: 10.1038/s41440-025-02392-9 (PMC12678181; doi:10.1038/s41440-025-02392-9)
Supplement: Supplementary file 1 — Supplementary Tables [file 41440_2025_2392_MOESM1_ESM.docx]

**Supplementary Table 1**. Partial correlations of selected biomarkers and risk factors in the total group

|  | WHtR | HDL-C | LDL-C | HbA1c | Smoking | Alcohol | MET-minutes/week | bSBP |
| --- | --- | --- | --- | --- | --- | --- | --- | --- |
| P-selectin | **r=0.075**  **p=0.010** | **r=0.088**  **p=0.003** | **r=0.160**  **p<0.001** | r=0.016  p=0.576 | r=-0.032  p=0.269 | **r=0.123**  **p<0.001** | r=-0.052  p=0.088 | **r=0.066**  **p=0.023** |
| PAI-1 | **r=0.387**  **p<0.001** | **r=-0.135**  **p<0.001** | **r=0.140**  **p<0.001** | **r=0.080**  **p=0.007** | **r=0.074**  **p=0.012** | r=0.054  p=0.066 | **r=-0.065**  **p=0.036** | **r=0.162**  **p<0.001** |
| GR | r=0.038  p=0.194 | **r=0.182**  **p<0.001** | **r=0.153**  **p<0.001** | **r=0.118**  **p<0.001** | r=-0.050  p=0.085 | **r=0.059**  **p=0.046** | r=-0.002  p=0.957 | **r=0.075**  **p=0.010** |
| ROS | **r=0.120**  **p<0.001** | **r=0.169**  **p<0.001** | **r=0.133**  **p<0.001** | **r=0.106**  **p<0.001** | r=0.054  p=0.064 | **r=0.088**  **p=0.003** | r=0.029  p=0.348 | **r=0.073**  **p=0.012** |
| IL-10 | **r=-0.076**  **p=0.010** | r=-0.013  p=0.652 | r=-0.054  p=0.067 | r=-0.052  p=0.078 | r=-0.045  p=0.126 | r=-0.033  p=0.255 | r=0.006  p=0.833 | r=-0.028  p=0.338 |
| IL-6 | **r=0.396**  **p<0.001** | **r=-0.282**  **p<0.001** | r=-0.030  p=0.308 | **r=0.109**  **p<0.001** | **r=0.059**  **p=0.042** | **r=-0.073**  **p=0.012** | **r=-0.060**  **p=0.049** | **r=0.100**  **p=0.001** |
| CRP | **r=0.460**  **p<0.001** | **r=-0.083**  **p=0.004** | **r=0.204**  **p<0.001** | **r=0.186**  **p<0.001** | r=0.029  p=0.325 | r=0.025  p=0.396 | **r=-0.064**  **p=0.036** | **r=0.119**  **p<0.001** |
| Fibrinogen | **r=0.378**  **p<0.001** | **r=-0.153**  **p<0.001** | **r=0.108**  **p<0.001** | **r=0.194**  **p<0.001** | r=-0.028  p=0.348 | r=-0.050  p=0.091 | r=-0.039  p=0.208 | **r=0.106**  **p<0.001** |
| MCP-1 | **r=0.107**  **p<0.001** | **r=-0.166**  **p<0.001** | r=-0.047  p=0.108 | r=0.037  p=0.207 | r=0.036  p=0.211 | r=-0.030  p=0.296 | r=0.013  p=0.679 | **r=0.073**  **p=0.012** |
| GDF-15 | r=-0.027  p=0.362 | **R=0.114**  **p<0.001** | **r=0.086**  **p=0.003** | **r=0.146**  **p<0.001** | **r=0.163**  **p<0.001** | **r=0.108**  **p<0.001** | r=-0.043  p=0.162 | r=0.026  p=0.369 |

Adjusted for age, sex and ethnicity.

Abbreviations: WHtR, waist-to-height ratio; HDL-C, high-density lipoprotein cholesterol; LDL-C, low-density lipoprotein cholesterol; HbA1c, glycated haemoglobin; MET, metabolic equivalent; bSBP, brachial systolic blood pressure; PAI-1, plasminogen activator inhibitor-1; GR, glutathione reductase; ROS, reactive oxygen species; IL-10, interleukin-10; IL-6, interleukin-6; CRP, C-reactive protein; MCP-1, monocyte chemoattractant protein 1; GDF-15, growth differentiation factor 15.

**Supplementary Table 2A**. Backward multiple regressions with different biomarkers as dependent variables and WHtR as main independent variable in the total group and the WHtR risk group

|  | **Total group** |  | **WHtR risk group** |  |
| --- | --- | --- | --- | --- |
|  | **β (95% CI)** | **p-value** | **β (95% CI)** | **p-value** |
| **Dependent variable: IL-6** | Model Adj R^2^=0.253, p<0.001 |  | Model Adj R^2^=0.299, p<0.001 |  |
| **WHtR** | 0.352 (0.295; 0.409) | <0.001 | 0.265 (0.181; 0.578) | <0.001 |
| Sex | -0.301 (-0.416; -0.187) | <0.001 | -0.481 (-0.773; -0.188) | 0.001 |
| Ethnicity | -0.298 (-0.428; -0.169) | <0.001 | - | - |
| SES score | -0.068 (-0.129; -0.006) | 0.031 | - | - |
| LDL-C | -0.074 (-0.133; -0.015) | 0.014 | -0.155 (-0.245; -0.015) | 0.027 |
| HDL-C | -0.162 (-0.223; -0.101) | <0.001 | -0.159 (-0.304; -0.015) | 0.031 |
| HbA1c | 0.074 (0.016; 0.132) | 0.012 | 0.213 (0.058; 0.278) | 0.003 |
| Smoking | 0.140 (0.020; 0.260) | 0.022 | - | - |
| Alcohol | -0.134 (-0.242; -0.025) | 0.016 | - | - |
| Total MET-minutes/week | -0.062 (-0.115; -0.008) | 0.025 | - | - |
| bSBP | - | - | -0.131 (-0.270; 0.012) | 0.073 |
|  |  |  |  |  |
| **Dependent variable: CRP** | Model Adj R^2^=0.305, p<0.001 |  | Model Adj R^2^=0.328, p<0.001 |  |
| **WHtR** | 0.416 (0.364; 0.467) | <0.001 | 0.267 (0.161; 0.480) | <0.001 |
| Sex | -0.467 (-0.567; -0.367) | <0.001 | -0.209 (-0.431; 0.013) | 0.065 |
| Ethnicity | -0.162 (-0.274; -0.049) | 0.005 | - | - |
| LDL-C | 0.108 (0.055; 0.161) | <0.001 |  |  |
| HbA1c | 0.120 (0.065; 0.175) | <0.001 | 0.248 (0.074; 0.253) | <0.001 |
| Smoking | - | - | -0.210 (-0.417; -0.002) | 0.048 |
| bSBP | - | - | -0.287 (-0.352; -0.122) | <0.001 |
|  |  |  |  |  |
| **Dependent variable: Fibrinogen** | Model Adj R^2^=0.284, p<0.001 |  | Model Adj R^2^=0.234, p<0.001 |  |
| **WHtR** | 0.321 (0.267; 0.375) | <0.001 | 0.233 (0.160; 0.646) | 0.001 |
| Sex | -0.713 (-0.822; -0.604) | <0.001 | -0.876 (-1.265; -0.486) | 0.000 |
| SES score | 0.049 (-0.004; 0.102) | 0.068 | 0.159 (0.001; 0.326) | 0.049 |
| HDL-C | -0.067 (-0.124; -0.011) | 0.020 | -0.198 (-0.422; -0.058) | 0.010 |
| HbA1c | 0.166 (0.113; 0.218) | <0.001 | - | - |
| Ethnicity | - | - | -0.445 (-0.815; -0.075) | 0.019 |
|  |  |  |  |  |
| **Dependent variable: PAI-1** | Model Adj R^2^=0.206, p<0.001 |  | Model Adj R^2^=0.240, p<0.001 |  |
| **WHtR** | 0.320 (0.259; 0.381) | <0.001 | - | - |
| Age | -0.081 (-0.138; -0.024) | 0.005 | - | - |
| Ethnicity | 0.380 (0.269; 0.491) | <0.001 | 0.247 (0.003; 0.491) | 0.047 |
| LDL-C | 0.098 (0.037; 0.159) | 0.002 | -0.139 (-0.198; 0.004) | 0.059 |
| HDL-C | -0.082 (-0.142; -0.022) | 0.008 | -0.171 (-0.266; -0.017) | 0.026 |
| Smoking | 0.186 (0.068; 0.304) | 0.002 | - | - |
| bSBP | 0.099 (0.042; 0.156) | 0.001 | - | - |
| Sex | - | - | 0.478 (0.220; 0.736) | <0.001 |
|  |  |  |  |  |
| **Dependent variable: GR** | Model Adj R^2^=0.091, p<0.001 |  | Model Adj R^2^=0.031, p=0.043 |  |
| **WHtR** | - | - | - | - |
| Age | 0.099 (0.039; 0.158) | 0.001 | - | - |
| Ethnicity | -0.124 (-0.254; 0.005) | 0.059 | - | - |
| LDL-C | 0.099 (0.037; 0.162) | 0.002 | - | - |
| HDL-C | 0.172 (0.111; 0.233) | <0.001 | 0.147 (-0.007; 0.372) | 0.059 |
| HbA1c | 0.109 (0.046; 0.172) | 0.001 | 0.146 (-0.008; 0.291) | 0.063 |
| Smoking | -0.120 (-0.245; 0.005) | 0.060 | - | - |
| bSBP | 0.089 (0.031; 0.148) | 0.003 | 0.146 (-0.010; 0.365) | 0.063 |
|  |  |  |  |  |
| **Dependent variable: ROS** | Model Adj R^2^=0.199, p<0.001 |  | Model Adj R^2^=0.269, p<0.001 |  |
| **WHtR** | 0.164 (0.108; 0.220) | <0.001 | 0.129 (-0.007; 0.432) | 0.058 |
| Sex | -0.517 (-0.631; -0.403) | <0.001 | -0.433 (-0.777; -0.089) | 0.014 |
| Ethnicity | -0.290 (-0.407; -0.173) | <0.001 | -0.444 (-0.763; -0.126) | 0.006 |
| HDL-C | 0.209 (0.150; 0.268) | <0.001 | 0.297 (0.180; 0.505) | <0.001 |
| HbA1c | 0.090 (0.031; 0.150) | 0.003 | - | - |
| Alcohol | 0.102 (-0.008; 0.212) | 0.070 | - | - |

Independent variables included in all models: age, sex, ethnicity, SES score, WHtR, HDL-C, LDL-C, HbA1c, smoking (categorical) alcohol (categorial), total MET-minutes physical activity/week, and bSBP.

- Independent variable did not contribute to the final regression model.

Abbreviations: WHtR, waist-to-height ratio; IL-6, interleukin-6; SES, socio-economic status; LDL-C, low-density lipoprotein cholesterol; HDL-C, high-density lipoprotein cholesterol; HbA1c, glycated haemoglobin; MET, metabolic equivalent; bSBP, brachial systolic blood pressure; CRP, C-reactive protein; PAI-1, plasminogen activator inhibitor-1; GR, glutathione reductase; ROS, reactive oxygen species.

**Supplementary Table 2B**. Backward multiple regressions with MCP-1 as dependent variable and HDL-C as main independent variable in the total group and the HDL-C risk group

|  | **Total group** |  | **HDL-C risk group** |  |
| --- | --- | --- | --- | --- |
|  | **β (95% CI)** | **p-value** | **β (95% CI)** | **p-value** |
| **Dependent variable: MCP-1** | Model Adj R^2^=0.198, p<0.001 |  | Model Adj R^2^=0.152, p<0.001 |  |
| **HDL-C** | -0.135 (-0.193; -0.077) | <0.001 | -0.091 (-0.330; -0.012) | 0.035 |
| WHtR | 0.070 (0.015; 0.125) | 0.012 | 0.093 (0.015; 0.153) | 0.017 |
| Sex | 0.385 (0.273; 0.498) | <0.001 | 0.348 (0.193; 0.504) | <0.001 |
| Ethnicity | -0.684 (-0.791; -0.577) | <0.001 | -0.618 (-0.759; -0.477) | <0.001 |

Independent variables included in model: age, sex, ethnicity, SES score, WHtR, HDL-C, LDL-C, HbA1c, smoking (categorical) alcohol (categorial), total MET-minutes physical activity/week, and bSBP.

Abbreviations: HDL-C, high-density lipoprotein cholesterol; MCP-1, monocyte chemoattractant protein 1; WHtR, waist-to-height ratio.

**Supplementary Table 2C**. Backward multiple regressions with P-selectin as dependent variable and LDL-C as main independent variable in the total group and the LDL-C risk group

|  | **Total group** |  | **LDL-C risk group** |  |
| --- | --- | --- | --- | --- |
|  | **β (95% CI)** | **p-value** | **β (95% CI)** | **p-value** |
| **Dependent variable: P-selectin** | Model Adj R^2^=0.096, p<0.001 |  | Model Adj R^2^=0.073, p<0.001 |  |
| **LDL-C** | 0.134 (0.069; 0.198) | <0.001 | - | - |
| HDL-C | 0.068 (0.000; 0.135) | 0.049 | - | - |
| WHtR | 0.065 (0.002; 0.127) | 0.041 | 0.097 (-0.017; 0.201) | 0.097 |
| Sex | 0.281 (0.157; 0.405) | <0.001 | 0.256 (0.017; 0.495) | 0.036 |
| Ethnicity | 0.406 (0.275; 0.537) | <0.001 | 0.460 (0.208; 0.712) | <0.001 |
| SES score | -0.079 (-0.146; -0.012) | 0.021 | - | - |
| Smoking | -0.155 (-0.288; -0.023) | 0.021 | -0.266 (-0.533; 0.001) | 0.051 |
| Alcohol | 0.231 (0.112; 0.350) | <0.001 | - | - |

Independent variables included in model: age, sex, ethnicity, SES score, WHtR, HDL-C, LDL-C, HbA1c, smoking (categorical) alcohol (categorial), total MET-minutes physical activity/week, and bSBP.

- Independent variable did not contribute to the final regression model.

Abbreviations: LDL-C, low-density lipoprotein cholesterol; HDL-C, high-density lipoprotein cholesterol; WHtR, waist-to-height ratio; SES, socio-economic status.

**Supplementary Table 2D**. Backward multiple regressions with GR as dependent variable and HbA1c as main independent variable in the total group and the HbA1c risk group

|  | **Total group** |  | **HbA1c risk group** |  |
| --- | --- | --- | --- | --- |
|  | **β (95% CI)** | **p-value** | **β (95% CI)** | **p-value** |
| **Dependent variable: GR** | Model Adj R^2^=0.091, p<0.001 |  | Model Adj R^2^=0.037, p=0.028 |  |
| **HbA1c** | 0.109 (0.046; 0.172) | 0.001 | - | - |
| HDL-C | 0.172 (0.111; 0.233) | <0.001 | 0.172 (0.002; 0.350) | 0.047 |
| Age | 0.099 (0.039; 0.158) | 0.001 | - | - |
| Ethnicity | -0.124 (-0.254; 0.005) | 0.059 | - | - |
| LDL-C | 0.099 (0.037; 0.162) | 0.002 | - | - |
| Smoking | -0.120 (-0.245; 0.005) | 0.060 | - | - |
| bSBP | 0.089 (0.031; 0.148) | 0.003 | - | - |
| WHtR | - | - | 0.197 (0.020; 0.274) | 0.024 |

Independent variables included in model: age, sex, ethnicity, SES score, WHtR, HDL-C, LDL-C, HbA1c, smoking (categorical) alcohol (categorial), total MET-minutes physical activity/week, and bSBP.

- Independent variable did not contribute to the final regression model.

Abbreviations: HbA1c, glycated haemoglobin; HDL-C, high-density lipoprotein cholesterol; LDL-C, low-density lipoprotein cholesterol; bSBP, brachial systolic blood pressure; WHtR, waist-to-height ratio.

**Supplementary Table 2E**. Backward multiple regressions with IL-10 and GDF-15 as dependent variables and smoking as main independent variable in the total group and the smoking risk group

|  | **Total group** |  | **Smoking risk group** |  |
| --- | --- | --- | --- | --- |
|  | **β (95% CI)** | **p-value** | **β (95% CI)** | **p-value** |
| **Dependent variable: IL-10** | Model Adj R^2^=0.016, p<0.001 |  | Model Adj R^2^=0.000, p= - |  |
| **Smoking** | - | - | - | - |
| WHtR | -0.069 (-0.129; -0.009) | 0.023 | - | - |
| Ethnicity | 0.233 (0.113; 0.352) | <0.001 | - | - |
|  |  |  |  |  |
| **Dependent variable: GDF-15** | Model Adj R^2^=0.088, p<0.001 |  | Model Adj R^2^=0.119, p<0.001 |  |
| **Smoking** | 0.278 (0.148; 0.408) | <0.001 | - | - |
| HbA1c | 0.163 (0.104; 0.221) | <0.001 | 0.141 (0.042; 0.275) | 0.008 |
| HDL-C | 0.115 (0.057; 0.174) | <0.001 | - | - |
| Age | 0.121 (0.058; 0.184) | <0.001 | 0.146 (0.038; 0.274) | 0.010 |
| SES score | -0.104 (-0.168; -0.039) | 0.002 | -0.231 (-0.355; -0.125) | 0.000 |
| Alcohol | 0.135 (0.016; 0.253) | 0.027 | 0.241 (0.003; 0.479) | 0.047 |
| WHtR | - | - | -0.192 (-0.298; -0.083) | 0.001 |
| LDL-C | - | - | 0.098 (-0.012; 0.212) | 0.081 |
| Total MET-minutes/week | - | - | -0.144 (-0.258; -0.038) | 0.008 |

Independent variables included in models: age, sex, ethnicity, SES score, WHtR, HDL-C, LDL-C, HbA1c, smoking (categorical) alcohol (categorial), total MET-minutes physical activity/week, and bSBP.

- Independent variable did not contribute to the final regression model.

Abbreviations: IL-10, interleukin-10; WHtR, waist-to-height ratio; GDF-15, growth differentiation factor 15; HbA1c, glycated haemoglobin; HDL-C, high-density lipoprotein cholesterol; SES, socio-economic status; LDL-C, low-density lipoprotein cholesterol; MET, metabolic equivalent.

**Supplementary Table 2F**. Backward multiple regressions with different biomarkers as dependent variables and alcohol use as main independent variable in the total group and the alcohol risk group

|  | **Total group** |  | **Alcohol risk group** |  |
| --- | --- | --- | --- | --- |
|  | **β (95% CI)** | **p-value** | **β (95% CI)** | **p-value** |
| **Dependent variable: PAI-1** | Model Adj R^2^=0.206, p<0.001 |  | Model Adj R^2^=0.244, p<0.001 |  |
| **Alcohol** | - | - | - | - |
| Smoking | 0.186 (0.068; 0.304) | 0.002 | 0.154 (-0.002; 0.310) | 0.053 |
| HDL-C | -0.082 (-0.142; -0.022) | 0.008 | -0.069 (-0.145; 0.009) | 0.083 |
| WHtR | 0.320 (0.259; 0.381) | <0.001 | 0.364 (0.286; 0.442) | <0.001 |
| Age | -0.081 (-0.138; -0.024) | 0.005 | -0.104 (-0.186; -0.031) | 0.006 |
| Ethnicity | 0.380 (0.269; 0.491) | <0.001 | 0.385 (0.237; 0.533) | <0.001 |
| LDL-C | 0.098 (0.037; 0.159) | 0.002 | - | - |
| bSBP | 0.099 (0.042; 0.156) | 0.001 | 0.122 (0.037; 0.209) | 0.005 |
| Sex | - | - | 0.231 (0.049; 0.414) | 0.013 |
|  |  |  |  |  |
| **Dependent variable: P-selectin** | Model Adj R^2^=0.096, p<0.001 |  | Model Adj R^2^=0.074, p<0.001 |  |
| **Alcohol** | 0.231 (0.112; 0.350) | <0.001 |  |  |
| Smoking | -0.155 (-0.288; -0.023) | 0.021 | -0.144 (-0.307; 0.018) | 0.082 |
| HDL-C | 0.068 (0.000; 0.135) | 0.049 |  |  |
| WHtR | 0.065 (0.002; 0.127) | 0.041 |  |  |
| Sex | 0.281 (0.157; 0.405) | <0.001 | 0.264 (0.108; 0.420) | 0.001 |
| Ethnicity | 0.406 (0.275; 0.537) | <0.001 | 0.427 (0.243; 0.611) | <0.001 |
| SES score | -0.079 (-0.146; -0.012) | 0.021 | -0.092 (-0.176; 0.002) | 0.057 |
| LDL-C | 0.134 (0.069; 0.198) | <0.001 | 0.130 (0.046; 0.194) | 0.002 |
|  |  |  |  |  |
| **Dependent variable: GDF-15** | Model Adj R^2^=0.088, p<0.001 |  | Model Adj R^2^=0.092, p<0.001 |  |
| **Alcohol** | 0.135 (0.016; 0.253) | 0.027 | - | - |
| Smoking | 0.278 (0.148; 0.408) | <0.001 | 0.315 (0.154; 0.475) | <0.001 |
| HbA1c | 0.163 (0.104; 0.221) | <0.001 | 0.178 (0.102; 0.265) | <0.001 |
| HDL-C | 0.115 (0.057; 0.174) | <0.001 | 0.128 (0.049; 0.200) | 0.001 |
| Age | 0.121 (0.058; 0.184) | <0.001 | 0.122 (0.036; 0.213) | 0.006 |
| SES score | -0.104 (-0.168; -0.039) | 0.002 | -0.116 (-0.200; -0.027) | 0.010 |

Independent variables included in all models: age, sex, ethnicity, SES score, WHtR, HDL-C, LDL-C, HbA1c, smoking (categorical) alcohol (categorial), total MET-minutes physical activity/week, and bSBP.

- Independent variable did not contribute to the final regression model.

Abbreviations: PAI-1, plasminogen activator inhibitor-1; HDL-C, high-density lipoprotein cholesterol; WHtR, waist-to-height ratio; LDL-C, low-density lipoprotein cholesterol; bSBP, brachial systolic blood pressure; SES, socio-economic status; GDF-15, growth differentiation factor 15; HbA1c, glycated haemoglobin.

**Supplementary Table 2G**. Backward multiple regressions with different biomarkers as dependent variables and physical activity (total MET-minutes/week) as main independent variable in the total group and the physical inactivity risk group

|  | **Total group** |  | **Physical inactivity risk group** |  |
| --- | --- | --- | --- | --- |
|  | **β (95% CI)** | **p-value** | **β (95% CI)** | **p-value** |
| **Dependent variable: CRP** | Model Adj R^2^=0.305, p<0.001 |  | Model Adj R^2^=0.233, p<0.001 |  |
| **Total MET-minutes/week** | - | - | 0.140 (-0.042; 0.698) | 0.082 |
| LDL-C | 0.108 (0.055; 0.161) | <0.001 | 0.136 (-0.022; 0.262) | 0.097 |
| WHtR | 0.416 (0.364; 0.467) | <0.001 | 0.376 (0.185; 0.462) | <0.001 |
| Sex | -0.467 (-0.567; -0.367) | <0.001 | -0.474 (-0.787; -0.162) | 0.003 |
| Ethnicity | -0.162 (-0.274; -0.049) | 0.005 | - | - |
| HbA1c | 0.120 (0.065; 0.175) | <0.001 | 0.157 (0.000; 0.317) | 0.050 |
|  |  |  |  |  |
| **Dependent variable: GDF-15** | Model Adj R^2^=0.088, p<0.001 |  | Model Adj R^2^=0.129, p<0.001 |  |
| **Total MET-minutes/week** | - | - | - | - |
| HDL-C | 0.115 (0.057; 0.174) | <0.001 | - | - |
| Age | 0.121 (0.058; 0.184) | <0.001 | - | - |
| SES score | -0.104 (-0.168; -0.039) | 0.002 | - | - |
| HbA1c | 0.163 (0.104; 0.221) | <0.001 | 0.191 (0.024; 0.354) | 0.025 |
| Smoking | 0.278 (0.148; 0.409) | <0.001 | 0.654 (0.303; 1.004) | <0.001 |
| Alcohol | 0.135 (0.016; 0.253) | 0.027 | - | - |
| Sex | - | - | -0.314 (-0.682; 0.055) | 0.094 |
| bSBP | - | - | 0.173 (-0.013; 0.332) | 0.070 |
|  |  |  |  |  |
| **Dependent variable: GR** | Model Adj R^2^=0.091, p<0.001 |  | Model Adj R^2^=0.138, p<0.001 |  |
| **Total MET-minutes/week** | - | - | - | - |
| LDL-C | 0.099 (0.037; 0.162) | 0.002 | - | - |
| HDL-C | 0.172 (0.111; 0.233) | <0.001 | 0.220 (0.052; 0.416) | 0.012 |
| Age | 0.099 (0.039; 0.158) | 0.001 | 0.160 (-0.012; 0.353) | 0.067 |
| Ethnicity | -0.124 (-0.254; 0.005) | 0.059 | - | - |
| HbA1c | 0.109 (0.046; 0.172) | 0.001 | 0.264 (0.104; 0.468) | 0.002 |
| Smoking | -0.120 (-0.245; 0.005) | 0.060 | - | - |
| bSBP | 0.089 (0.031; 0.148) | 0.003 | - | - |

Independent variables included in all models: age, sex, ethnicity, SES score, WHtR, HDL-C, LDL-C, HbA1c, smoking (categorical) alcohol (categorial), total MET-minutes physical activity/week, and bSBP.

- Independent variable did not contribute to the final regression model.

Abbreviations: CRP, C-reactive protein; MET, metabolic equivalent; LDL-C, low-density lipoprotein cholesterol; WHtR, waist-to-height ratio; HbA1c, glycated haemoglobin; GDF-15, growth differentiation factor 15; HDL-C, high-density lipoprotein cholesterol; SES, socio-economic status; bSBP, brachial systolic blood pressure; GR, glutathione reductase.

**Supplementary Table 2H**. Backward multiple regressions with different biomarkers as dependent variables and bSBP as main independent variable in the total group and the blood pressure risk group

|  | **Total group** |  | **Blood pressure risk group** |  |
| --- | --- | --- | --- | --- |
|  | **β (95% CI)** | **p-value** | **β (95% CI)** | **p-value** |
| **Dependent variable: IL-10** | Model Adj R^2^=0.016, p<0.001 |  | Model Adj R^2^=0.038, p=0.001 |  |
| **bSBP** | - | - | 0.168 (0.077; 0.382) | 0.003 |
| WHtR | -0.069 (-0.129; -0.009) | 0.023 | - | - |
| Ethnicity | 0.233 (0.113; 0.352) | <0.001 | 0.216 (-0.008; 0.440) | 0.058 |
|  |  |  |  |  |
| **Dependent variable: CRP** | Model Adj R^2^=0.305, p<0.001 |  | Model Adj R^2^=0.326, p<0.001 |  |
| **bSBP** | - | - | -0.084 (-0.241; 0.019) | 0.094 |
| LDL-C | 0.108 (0.055; 0.161) | 0.000 | 0.161 (0.061; 0.259) | 0.002 |
| WHtR | 0.416 (0.364; 0.467) | 0.000 | 0.340 (0.211; 0.393) | 0.000 |
| Sex | -0.467 (-0.567; -0.367) | 0.000 | -0.491 (-0.705; -0.277) | 0.000 |
| Ethnicity | -0.162 (-0.274; -0.049) | 0.005 | - | - |
| HbA1c | 0.120 (0.065; 0.175) | 0.000 | 0.163 (0.066; 0.248) | 0.001 |
|  |  |  |  |  |
| **Dependent variable: GR** | Model Adj R^2^=0.091, p<0.001 |  | Model Adj R^2^=0.061, p<0.001 |  |
| **bSBP** | 0.089 (0.031; 0.148) | 0.003 | - | - |
| LDL-C | 0.099 (0.037; 0.162) | 0.002 | - | - |
| HDL-C | 0.172 (0.111; 0.233) | 0.000 | 0.233 (0.131; 0.368) | 0.000 |
| Age | 0.099 (0.039; 0.158) | 0.001 | - | - |
| Ethnicity | -0.124 (-0.254; 0.005) | 0.059 | - | - |
| HbA1c | 0.109 (0.046; 0.172) | 0.001 | - | - |
| Smoking | -0.120 (-0.245; 0.005) | 0.060 | - | - |
| WHtR | - | - | 0.152 (0.040; 0.255) | 0.007 |

Independent variables included in all models: age, sex, ethnicity, SES score, WHtR, HDL-C, LDL-C, HbA1c, smoking (categorical) alcohol (categorial), total MET-minutes physical activity/week, and bSBP.

- Independent variable did not contribute to the final regression model.

Abbreviations: IL-10, interleukin-10; bSBP, brachial systolic blood pressure; WHtR, waist-to-height ratio; CRP, C-reactive protein; LDL-C, low-density lipoprotein cholesterol; HbA1c, glycated haemoglobin; GR, glutathione reductase; HDL-C, high-density lipoprotein cholesterol.
